# Chest wall-necrotizing soft tissue infection caused by *Finegoldia magna* and *Helcococcus kunzii* in an immunocompromised patient: a case report

**DOI:** 10.1128/asmcr.00046-24

**Published:** 2024-12-10

**Authors:** Thamer Alaifan, Abdualhakeem Al-Thaqafi, Enas Munshi, Mai Kaaki, Abdulrahman Khojah, Abdulrazak Sakhakhni

**Affiliations:** 1 Department of Adult Critical Care Medicine, Ministry of National Guard—Health Affairs, Jeddah, Saudi Arabia; 2 College of Medicine, King Saud bin Abdulaziz University for Health Sciences, Jeddah, Saudi Arabia; 3 King Abdullah International Medical Research Center, Jeddah, Saudi Arabia; 4 Department of Infectious Disease, Ministry of National Guard—Health Affairs, Jeddah, Saudi Arabia; 5 Department of Microbiology, Ministry of National Guard—Health Affairs, Jeddah, Saudi Arabia; Vanderbilt University Medical Center, Nashville, Tennessee, USA

**Keywords:** necrotizing soft tissue infections, *Finegoldia magna*, *Helcococcus kunzii*, case report

## Abstract

**Background:**

Necrotizing soft tissue infections are life-threatening conditions that require prompt diagnosis and intervention. This case report presents a rare case of chest wall-necrotizing soft tissue infection caused by *Finegoldia magna* and *Helcococcus kunzii* in an immunocompromised patient.

**Case Summary:**

A 60-year-old female with metastatic uterine leiomyosarcoma and multiple comorbidities presented with swelling of the right breast and discharge. Surgical intervention revealed extensive necrosis, and cultures identified *Finegoldia magna* and *Helcococcus kunzii*. Despite antibiotic therapy and repeated debridement, the patient developed septic shock and coagulopathy, ultimately succumbing to infection.

**Conclusion:**

This case highlights the potential of rare organisms to cause severe necrotizing soft tissue infections in immunocompromised individuals. Rare microbiological and anatomical presentations and findings are interesting incidents that require further attention. It is important to note that, although these organisms are rare, they still pose a significant risk for the development of necrotizing soft tissue infections. By reporting such cases, we will aid in raising awareness of these organisms.

## INTRODUCTION

Necrotizing soft tissue infections (NSTIs) are aggressive, rapidly progressive, and life-threatening deep-tissue infections (1, 2). The variability in presenting signs and symptoms linked to NSTI represents a diagnostic challenge (3, 4). However, early and accurate diagnosis is crucial for successful treatment (1, 2). The Laboratory Risk Indicator for Necrotizing Fasciitis (LRINEC) score was developed to evaluate the severity and prognosis of NSTI, although its validity has been questioned (5, 6). The standard approach is early and extensive debridement to reach viable soft tissue with delayed repair and wound closure combined with appropriate antibiotics (7). Despite advances in medical care, the mortality rate remains between 25% and 35% (1, 7, 8).

*Finegoldia magna* (*F. magna*), previously known as *Peptostreptococcus magnus*, is a member of the *Clostridiales* family and a pleomorphic Gram-positive, non-spore-forming obligate anaerobe (9). Isolation and identification of this organism relies on phenotypic methods and biochemical testing (9). *F. magna* is a major anaerobic commensal; however, it is often underestimated as a significant pathogen (9). *F. magna* has been reported to be associated with necrotizing pneumonia (10, 11), native and prosthetic valve endocarditis (12, 13), pericarditis and mediastinitis (14, 15), NSTI (16–19), and native and prosthetic joint septic arthritis (10, 20).

*Helcococcus kunzii* (*H. kunzii*) is a Gram-positive, facultative, catalase-negative, non-sporulating, anaerobic coccus that was first identified as a commensal bacterium of the human skin (21, 22). However, the significance of *H. kunzii* infection is yet to be understood (21, 22). Its detection can be challenging because it is often isolated from mixed cultures and may be overgrown by other microorganisms (21, 22). Later, it was shown that biochemical isolation methods are unreliable and have mixed features with other bacterial organisms (21–26).

To our knowledge, this is the first documented case of *F. magna* and *H. kunzii* causing chest wall NSTI in an immunocompromised patient. We present similar cases of *F. magna* and *H. kunzii* and a review of the two organisms.

## CASE PRESENTATION

A 60-year-old female is in supportive care with a history of uterine leiomyosarcoma stage IV with lung metastasis. She had undergone total abdominal hysterectomy and bilateral salpingo-oophorectomy (TAH-BSO) 2 years prior to presentation. She received palliative radiotherapy and chemotherapy (gemcitabine and docetaxel); the last dose was 1 month prior to her presentation. Her oncological course was complicated by obstructive uropathy, which required bilateral nephrostomy tubes that were changed 6 months prior to her presentation. Additionally, she had a medical history of hypertension, type-2 diabetes mellitus (DM), and chronic hepatitis C virus infection.

She presented to the emergency department (ED) complaining of a swollen right breast with purulent discharge. The swelling started as a small lump 7 months before her presentation and grew more rapidly and painfully over the 3 weeks preceding her presentation. She denied any history of fever, weight loss, night sweats, or loss of appetite. The review of other organ systems was normal. She also denied recent contact with animals or trauma. Physical examination revealed a blood pressure of 97/63 mmHg, heart rate of 100 beats per minute, respiratory rate of 21 breaths per minute, temperature of 37.1°C, and oxygen saturation of 98% in room air.

Breast examination revealed a swollen red carbuncle measuring 3 × 3 in the lower quadrants of her right breast with pus discharge. However, the nipple and surrounding skin were intact. Additionally, her left breast and bilateral axilla were intact. The remainder of her systemic examination, including cardiopulmonary, gastrointestinal, and genitourinary examinations, was unremarkable.

Laboratory parameters at presentation revealed hemoglobin (Hgb) of 8.7 g/dL, white blood cells (WBC) of 9.3 × 10^9^/L, platelets of 312 × 10^9^/L, and C-reactive protein of 174 mg/L. The LRINEC score was 8 at the time of presentation. The emergency physician referred the patient to the surgical team for drainage of the abscess. She was admitted under the surgical team and taken to the operating room for urgent abscess drainage. Multiple pockets were found, and all were drained. Necrotic tissue was found underneath the superficial pockets. Debridement was expanded further and further down for almost two-thirds of the right breast, with multiple attempts to reach healthy tissues. However, the infection and necrosis were extensive beyond the breast tissue. Cultures and pathology samples were obtained from deep necrotic tissue. She was started concurrently on piperacillin–tazobactam 4.5 g four times a day empirically. Compromising the chest wall configuration was a major concern for the surgical team due to the unexpected deep-seated abscesses, and the patient was planned for a further operation.

Two cultures were obtained from the initial operation, which were finalized 2 days post-operation. One culture was positive for *F. magna* and *H. kunzii*, and the other yielded a heavy growth of *H. kunzii*. The media workup for *F. magna* showed no growth on sheep blood and MacConkey agars after 2 days, slight turbidity in cooked meat, and the presence of anaerobes on neomycin blood agar after 2 days. Identification was performed using the matrix-assisted laser desorption/ionization-time of flight (MALDI-TOF) technique, with the Vitek MS result confirming *F. magna* at 99.9%. Susceptibility was determined using the Kirby–Bauer technique. The results showed sensitivity to vancomycin and clindamycin but resistance to metronidazole. The media workup for *H. kunzii* showed the presence of *Streptococcus* on sheep blood agar and neomycin blood agar after 2 days, turbidity in cooked meat, and no growth on chocolate agar, MacConkey agar, or Sabouraud agar after 2 days. Identification was performed using the MALDI-TOF technique, with Vitek MS results confirming *Helcococcus kunzii* at 99.9%. Susceptibility was determined using the Kirby–Bauer technique, and manual interpretation was performed using zone size under the streptococcal family. The results showed sensitivity to vancomycin, penicillin, clindamycin, and ampicillin. The references were set according to the M100 Performance Standards for Antimicrobial Susceptibility Testing (27).

Two days later, the patient’s wound had a discharge from the surgical site, and she was hypotensive with no response to fluids. The patient’s condition progressed, and she was unstable for further investigation and imaging. She underwent a second operation, in which the surgeons found pus discharge with extended necrotic tissue underneath. Excision attempts failed to reach healthy tissues, as the necrosis was deep and beyond the breast tissues. The patient was transferred to the intensive care unit post-operation. The patient showed signs of septic shock and coagulopathy. She was started on two inotropes, and her antibiotics escalated to vancomycin 1 g every 24 h and clindamycin 900 mg every 24 h, according to the sensitivity. The patient’s underlying condition and active malignancy hindered the recovery and treatment process. The family was updated and informed regarding the patient’s condition. Unfortunately, the patient passed away secondary to septic shock secondary to necrotizing soft tissue infection.

## DISCUSSION

This is a case of an immunocompromised patient with chest wall NSTI caused by two rare bacterial organisms, *F. magna* and *H. kunzii*. Searching the database for cases of NSTI caused by *F. magna* revealed two abdominal wall NSTI in diabetic patients (16, 17), NSTI secondary to diabetic foot (18), polymicrobial, *F. magna* included, NSTI secondary to dog bite in a diabetic lady (19), and breast abscess (28). All cases were treated with excision of necrotic tissue and antibiotics (16–18). One study analyzed 222 patients and found evidence of mixed infections involving *F. magna* in 151 cases and pure cultures of *F. magna* in 32 cases (29). Another study investigating Gram-positive anaerobic cocci in a cancer center reported dominant *F. magna* isolates, most commonly from surgical wounds (45%), body fluids (29%), and abscesses (24%) (30).

To the best of our knowledge, no cases of NSTI associated with *H. kunzii* have been reported. The genus *Helcococcus* is uncommon in humans. One case reported an immunocompetent patient presenting with a breast abscess secondary to *H. kunzii* isolated by 16S rDNA PCR (31). In another study, *H. kunzii* was detected via 16S rDNA PCR in a case of culture-negative infective endocarditis (32). Earlier studies have questioned the pathogenicity of *H. kunzii*, which was treated as colonization or contamination (33, 34). Later studies reported that *H. kunzii* was isolated as the sole organism in several clinical contexts, and it was resolved after therapeutic interventions (21, 22, 24, 25, 35). In our case and other reported cases, using the MALDI-TOF technique improved the detection of such organism (22, 35, 36).

In our case, the patient had an underlying malignancy, an underlying breast lump, DM, and she received docetaxel chemotherapy. According to the classification of NSTI by predisposing factors, our patient presented with class I NSTI (37). Moreover, a study noted that docetaxel-based chemotherapy is related to non-neutropenic infections, partly due to CD4+ lymphopenia (38). Chest wall NSTIs are particularly rare and severe due to the interplay of factors, including the rate of progression of the infection, the extent of tissue damage, and the overall state of the patient (39, 40).

Reporting such unusual microbes adds to the current literature regarding their detection and treatment. Early suspicion and intervention are the mainstay in clinical practice.

## Data Availability

All related data are presented in this article.
